# Systematic review of community-based, school-based, and combined delivery modes for reaching school-aged children in mass drug administration programs for schistosomiasis

**DOI:** 10.1371/journal.pntd.0006043

**Published:** 2017-10-27

**Authors:** Michael Burnim, Julianne A. Ivy, Charles H. King

**Affiliations:** 1 Center for Global Health and Diseases and WHO Collaborating Centre for Research and Training for Schistosomiasis Elimination, Case Western Reserve University School of Medicine, Cleveland, Ohio, United States of America; 2 Schistosomiasis Consortium for Operational Research and Evaluation, University of Georgia, Athens, Georgia, United States of America; University of Queensland, AUSTRALIA

## Abstract

**Background:**

The mainstay of current schistosomiasis control programs is mass preventive chemotherapy of school-aged children with praziquantel. This treatment is delivered through school-based, community-based, or combined school- and community-based systems. Attaining very high coverage rates for children is essential in mass schistosomiasis treatment programs, as is ensuring that there are no persistently untreated subpopulations, a potential challenge for school-based programs in areas with low school enrollment. This review sought to compare the different treatment delivery methods based both on their coverage of school-aged children overall and on their coverage specifically of non-enrolled children. In addition, qualitative community or programmatic factors associated with high or low coverage rates were identified, with suggestions for overall coverage improvement.

**Methodology/Principal findings:**

This review was registered prospectively with PROSPERO (CRD 42015017656). Five hundred forty-nine publication of potential relevance were identified through database searches, reference lists, and personal communications. Eligible studies included those published before October 2015, written in English or French, containing quantitative or qualitative data about coverage rates for MDA of school-aged children with praziquantel. Among the 22 selected studies, combined community- and school-based programs achieved the highest median coverage rates (89%), followed by community-based programs (72%). School-based programs had both the lowest median coverage of children overall (49%) and the lowest coverage of the non-enrolled subpopulation of children. Qualitatively, major factors affecting program success included fear of side effects, inadequate education about schistosomiasis, lack of incentives for drug distributors, and inequitable distribution to minority groups.

**Conclusions/Significance:**

This review provides an evidence-based framework for the development of future schistosomiasis control programs. Based on our results, a combined community and school-based delivery system should maximize coverage for both in- and out-of-school children, especially when combined with interventions such as snacks for treated children, educational campaigns, incentives for drug distributors, and active inclusion of marginalized groups.

**Trial registration:**

ClinicalTrials.gov CRD42015017656

## Author summary

Schistosomiasis is a chronic inflammatory condition, caused by parasitic flukes, that affects over 290 million people worldwide. Consequences of infection include anemia, stunted growth, liver abnormalities, and subfertility. Currently, the main approach to schistosomiasis control involves mass preventive treatment of school-aged children in endemic areas. The treatment, praziquantel, can be distributed through school-based, community-based, or combined school- and community-based systems. The first part of this systematic review compared the three delivery modes and found that combined delivery resulted in the best overall coverage of school-aged children, with community-only delivery the next-best approach. School-only delivery not only had the lowest overall coverage, but especially fell behind in targeting children not enrolled in school. As a whole, these results support the more frequent use of a combined approach to delivery in order to achieve the highest coverage rates and ensure that out-of-school children are not left persistently untreated. In the second part of this review the qualitative factors affecting program success were examined. The results indicate that overall treatment coverage can be improved via small interventions, such as snacks for participating children to reduce drug side effects, educational campaigns about schistosomiasis, incentives for drug distributors, and active inclusion of marginalized groups.

## Introduction

The Schistosomiasis Consortium for Operational Research and Evaluation (SCORE) was established in December 2008 to answer strategic questions about schistosomiasis control and elimination [1]. SCORE’s goal is to find answers that will help current and future schistosomiasis control program managers to do their job better, with its main focus being control of *Schistosoma haematobium* and *S*. *mansoni* infections in sub-Saharan Africa. This includes learning what approaches to controlling and eliminating schistosomiasis work best, and developing and evaluating new tools for program managers to use. SCORE research is intended inform efforts to gain control of schistosomiasis in high-prevalence areas, to sustain control and move towards elimination in areas of moderate prevalence, and ultimately to eliminate schistosomiasis. As part of the SCORE program, the present systematic review was undertaken to improve understanding of the factors that affect an individual’s adherence to treatment for schistosomiasis where large-scale mass drug administration (MDA) programs are implemented.

Schistosomiasis is a disease caused by water-borne, parasitic trematodes of *Schistosoma* species that infect either the genitourinary tract or the intestines and liver. Sequelae of chronic *Schistosoma* infection include anemia, malnutrition, growth retardation, poor school performance, infertility, and potentially fatal complications such as portal hypertension, renal failure, and bladder cancer [2]. Globally, more than 290 million individuals are estimated to be infected, with another 600–780 million at risk [3, 4]. The most prevalent species, *Schistosoma haematobium*, is found predominantly in sub-Saharan Africa and the Middle East, where it is transmitted by *Bulinus* species snails. *S*. *mansoni* is transmitted in Africa and South America via *Biomphalaria* species, and the third most prevalent parasite species, *S*. *japonicum*, is transmitted in East Asia and in the Philippines by *Oncomelania* species snails [1]. Because of SCORE’s focus on schistosome species found in sub-Saharan Africa, results for *S*. *japonicum* were not included in this review.

Historically, schistosomiasis control has employed a number of methods that limit the impact of disease. Approaches have included molluscicides that target the parasite’s intermediate snail host [5]; interventions to provide clean water, sanitation, and hygiene [6]; and selective or mass treatment with anti-schistosomal agents [7, 8]. In the past few decades, control efforts have primarily focused on the use of praziquantel for mass preventive chemotherapy of school-aged children (SAC) and other high-risk adult groups (e.g., fishermen, car washers, laundry workers) in endemic areas, through the use of MDA [7, 8].

A variety of MDA methods are currently used for schistosomiasis control. Delivery modes can broadly be divided into community-based and school-based programs, or a combination of the two. Community-based distribution strategies include house-to-house distribution, distribution from a health facility or other central location, or a combination of the two. Other program characteristics that may vary from study to study include: i) which personnel distribute the drugs, ii) how communities are educated and mobilized, and iii) whether the anti-schistosomal programs are “vertical” or combined with community-based campaigns against lymphatic filariasis and onchocerciasis.

With respect to school-based programs, most countries affected by schistosomiasis have significant percentages of SAC who are not enrolled in school, and not all children who are enrolled regularly attend school. From data collected between 2000 and 2006, UNICEF reported that in Eastern/Southern Africa, only 70% of children attended primary school, and that in West/Central Africa, only 62% of children attended primary school [9]. If school-based programs leave these sizeable populations of un-enrolled children persistently untreated, this will significantly impede the success of schistosomiasis control and elimination programs.

World Health Organization (WHO) guidelines recommend that in all endemic areas, at least 75% of children are treated either annually (in high endemicity zones with ≥ 50% SAC prevalence), every two years (in moderate endemicity zones having 10–49% SAC prevalence), or upon entering and leaving primary school (in low endemicity zones having < 10% SAC prevalence) [8]. Modeling has suggested that, due to the high risk of infection resurgence upon cessation of control programs [10–12], an even higher level of population coverage may be required for the effects of preventive chemotherapy to be sustained. For example, it is estimated that in “high-risk” villages (>50% SAC prevalence at baseline), greater than 90% coverage of SAC will be required in order to achieve 3–4 subsequent years of sustained low prevalence (i.e., at < 10%) [13]. With these coverage target numbers in mind, an understanding of what features contribute to the most successful MDA programs is essential.

This review sought to assess the overall SAC coverage rates as well as coverage rates of non-enrolled children obtained by MDA through respective school, community, or a combination of school and community delivery systems. We discuss the factors believed to be associated with high or low coverage rates, and summarize different national and regional programs’ suggestions for improving overall SAC coverage.

## Methods

### Ethics statement

The data used in this project were aggregated, anonymized data from previously published studies; as such, this study does not constitute human subjects research according to U.S. Department of Health and Human Services guidelines (https://www.hhs.gov/ohrp/regulations-and-policy/guidance).

### Study protocol and registration

The protocol for this study was developed prospectively by the authors. It was registered and published in the International Prospective Register of Systematic Reviews (PROSPERO) online database, https://www.crd.york.ac.uk/PROSPERO/#index.php, number CRD42015017656, on 06 April 2015. A copy of the registered protocol is found in S2 File.

### Eligibility criteria

In order to evaluate the effectiveness of MDA programs for schistosomiasis, we aimed to include any studies published before October 2015 that reported quantitative or qualitative data about coverage rates of SAC for MDA with praziquantel in sub-Saharan Africa, or in other low- or middle-income countries in the middle-east or South America. Articles in English or French were included. Relevant ‘gray’ literature (project reports, white papers) was also obtained and reviewed when possible.

### Information sources

The National Library of Medicine’s MEDLINE, along with Elsevier’s EMBASE, Google Scholar, African Journals Online, and Web of Science were used to identify published studies. The reference lists of these identified papers were then used to search for additional unindexed reports. When relevant, attempts were made to contact authors to clarify information and to obtain unpublished literature.

### Search strategy

Literature searches were performed using combinations of the following key words: ‘schistosomiasis’, ‘mansoni’, ‘haematobium/haematobia’, ‘prevention’, ‘control’, ‘compliance’, ‘adherence’, ‘uptake’, ‘coverage’, ‘non-enrolled’, ‘non-attendance’, ‘community-directed treatment’, and ‘MDA’. A search log containing citation information for each study was kept using Microsoft Excel.

### Study selection

The titles and abstracts of all studies identified in the literature search were screened for relevance to the present systematic review. The full texts of the studies not excluded after the screening phase of the study were obtained from online or library sources when possible. These reports were then reviewed in full to determine eligibility for the present systematic review.

### Data collection

Data from the included papers was abstracted and indexed in a Microsoft Access database. Besides citation information and year of publication, information was collected on study location, study design, MDA delivery method (community-based, school-based, or combined), and population characteristics. The fraction of all eligible SAC treated, the fraction of non-enrolled versus enrolled SAC treated, and the methods used to measure the number of eligible and treated individuals (e.g. censuses, household surveys, drug distributor registers) were also recorded. When a study reported coverage rates using both distributor registers and household surveys, the household survey coverage rate was used. Qualitative data extracted included factors positively or negatively associated with success of MDA, as well as reported side effects of the medication.

### Summary measures

Percentage of SAC coverage (including enrolled and non-enrolled children) was the primary measure used to compare methods of delivery of MDA; when available, coverage specifically of non-enrolled children was also compared between studies. Due to the heterogeneity in which studies reported MDA coverage, it was necessary to use multiple strategies to generate data that would be comparable for analysis: i) when an article reported an aggregate coverage rate for SAC and adults, the coverage rate of SAC was assumed to be at least that of the reported aggregate coverage rate; ii) when an article reported coverage rates by individual district, the coverage rate extracted was a weighted average of the individual district averages based on targeted population size; iii) when coverage rates were reported for multiple years, these were averaged and population-based weighting was again used, where appropriate, in calculating these blended averages.

Due to the heterogeneity of study sizes as well as methods of measuring coverage, it was not possible to perform formal meta-analyses, i.e., no summary point estimates or confidence intervals have been calculated, nor have statistical comparisons been made among or between distribution methods.

Qualitative analysis was performed to determine other factors, apart from method of delivery, that were commonly cited as affecting coverage rates in the included studies.

### Risk of bias across studies

The studies reviewed in this paper were mostly not protocol-driven randomized trials. They were primarily one-group observational, or pre- vs. post-modification evaluation trials, and they generally did not report on aspects of the study that might help to determine risk of bias. Publication bias could potentially have influenced data availability if reports of innovative MDA strategies were published only if successful, or if standard implementation programs were reported only if unsuccessful in reaching coverage targets.

## Results

### Study selection

**Fig 1:**
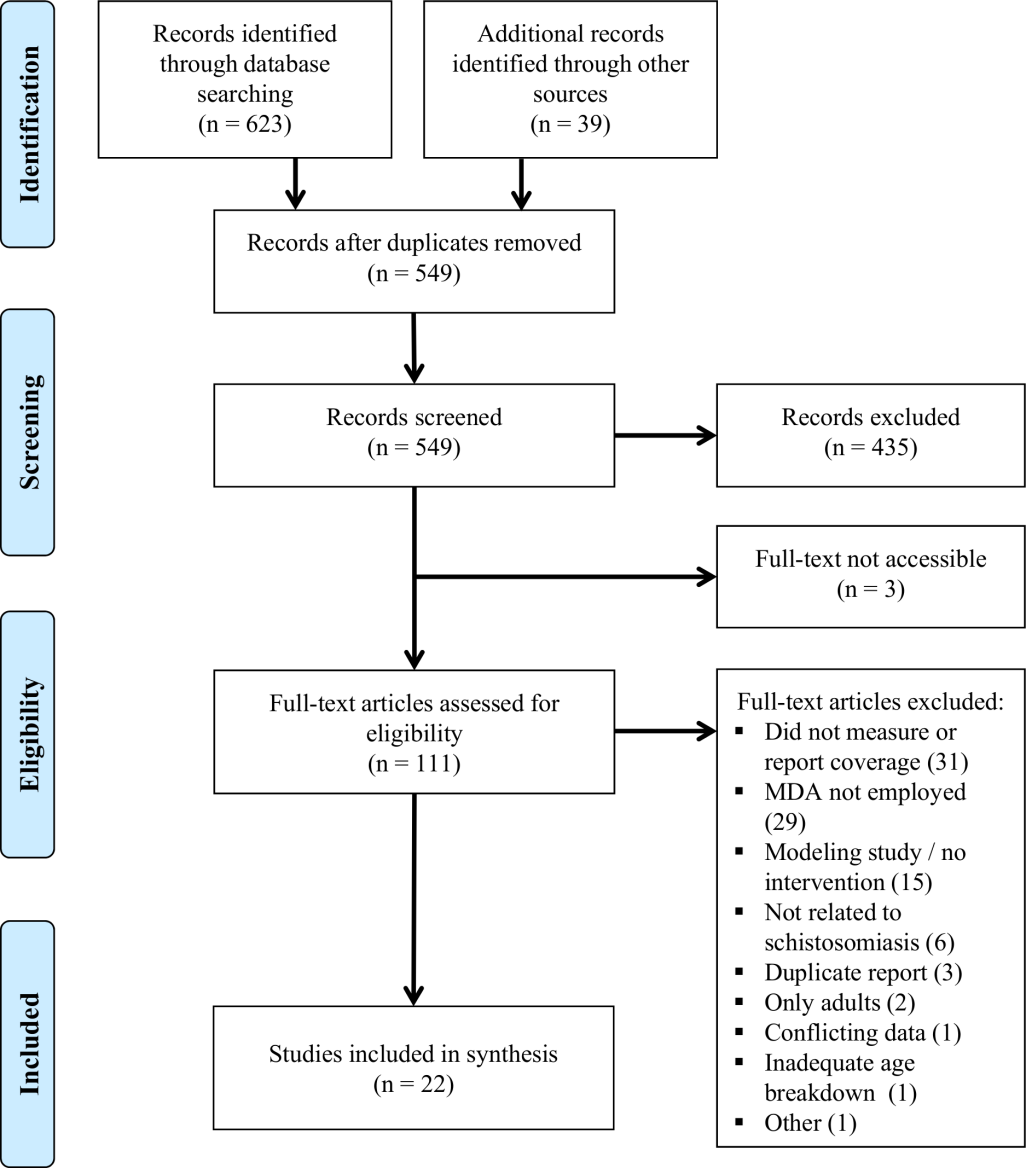
Flow chart for study selection. The flow diagram indicates the numbers of titles and studies reviewed in preparation of the current systematic review of treatment coverage effects of different approaches to MDA for schistosomiasis.

**Fig 1** shows a flow diagram of the study selection process for this systematic review. 623 references were identified through systematic database searches and 39 more through reference lists and personal communications. After screening of titles and abstracts, 114 papers were selected for full-text review; however, the full text could only be accessed for 111. **S1 Text** contains a list of the studies considered for full review. Reasons for exclusion (n = 89) included: did not measure or report coverage (31), MDA not employed (29), modeling study/no intervention (15), not schistosomiasis-related (6), duplicate report (3), only adults included (2), conflicting data (1), inadequate age category breakdown (1), or other (1).

### Study characteristics

Twenty-two studies were included in the systematic review. Fourteen of those studies (Table 1) reported coverage rates for populations of, or including, SAC. Of those fourteen, five reported on combined community- and school-based delivery, four reported on community-based delivery, two reported on school-based delivery, and three compared school-based and community-based delivery. Three of the quantitative studies also reported separate coverage rates for enrolled and non-enrolled children. Several of the studies reporting overall coverage rates, plus eight others (Table 2), provided qualitative analysis of individual, community, or programmatic factors influencing coverage.

**Table 1:** Included studies with quantitative data on MDA coverage rates.

Intervention	Study [reference]	Study Design (N)^a^	Country	Outcome Measurement Methods	Integrated Program?^b^	Coverage related factors discussed?
**Combined school- and community-based**	Oshish 2011 [14]	Pre-Post^c^ (325,509)	Yemen	Drug distributor registers and projected census data	No	Yes
	Gabrielli 2006 [15]	Pre-post (3,659,211)	Burkina Faso	School and drug distributer registers and census data	No	Yes
	Sesay 2014 [16]	Pre-Post (2,375,539)	Sierra Leone	Drug distributors registers and independent survey	No	No
	Dembélé 2012 [17]	Pre-post (3,046,078)	Mali	Drug distributor registers and census data	Yes	Yes
	Leslie 2011 [18]	Pre-Post (288,078)	Niger	Aggregate^d^ treatment registers and national annual treatment summary	No	No
**Community-based**	Hopkins 2002 [19]	Pre-post (5,214)	Nigeria	Not reported	Yes	No
	Anto 2011 [20]	Non-randomized control trial (3,520)	Ghana	Aggregate^d^ drug distributor registers	Yes	Yes
	Mwinzi 2012 [21]	Pre-post (3,677)	Kenya	Aggregate^d^ distributor registers and household surveys	No	Yes
	Chami 2015 [22]	Observational (935)	Uganda	Drug distributor registers, post-treatment surveys in villages	No	Yes
**School-based**	Muhumuza 2013a [23]	Observational (1,010)	Uganda	School-based survey of enrolled SAC	No	Yes
	Muhumuza 2013b [24]	Pre-post (1,020)	Uganda	School-based survey of enrolled SAC	No	Yes
**School-based vs. community-based**	Ndyomugyeni 2003 [25]	Non-randomized comparative trial (954)	Uganda	Household survey	Yes	Yes
	Mafe 2005 [26]	Non-randomized comparative trial (3,827)	Nigeria	Household survey	No	Yes
	Massa 2009 [27, 28]	Randomized comparative trial (7,039)	Tanzania	Drug distributor registers	No	Yes

^a^ N is overall number of targeted SAC in study

^b^ Programs were considered to be “integrated” if they included distribution of drugs other than PZQ and albendazole or mebendazole, such as ivermectin or DEC for onchocerciasis and filariasis.

^c^ Pre-post indicates outcomes were scored based on pre- and post-intervention surveys of a non-randomized trial to improve participation

^d^ Aggregate coverage rates were reported for adult and SAC, together, in these studies

**Table 2:** Studies describing qualitative factors influencing coverage.

Intervention	Study	Study Design	Country	Methods	Integrated^a^
**Combined school- and community-based**	Fleming 2009 [29]	Observational	Uganda	Key informant interviews and focus group discussions (FGDs)	No
	Kabatereine 2006 [30]	Pre-post^b^	Uganda	Key informant interviews	No
**Community-based**	Dabo 2013 [31]	Pre-post	Mali	Drug distributor registers, census, household surveys, FGDs, interviews	No
	Omedo 2012 [32]	Observational	Kenya	Unstructured group discussions	No
	Omedo 2014 [33]	Pre-post	Kenya	Semi-structured group discussions with drug distributers	No
**School-based**	Muhumuza 2014 [34]	Randomized comparative trial	Uganda	Interviews of children	No
	Muhumuza 2015 [35]	Observational	Uganda	Key informant interviews and FGDs	No
**School- based vs. community-based**	Massa 2009c [36]	Observational	Tanzania	In-depth interviews and FGDs	No

^a^ Programs considered to be “integrated” if they included distribution of drugs other than PZQ and albendazole or mebendazole

^b^ Pre-post indicates outcomes were scored based on pre- and post-intervention surveys of a non-randomized trial to improve participation

### Coverage rates by delivery method

**Fig 2:**
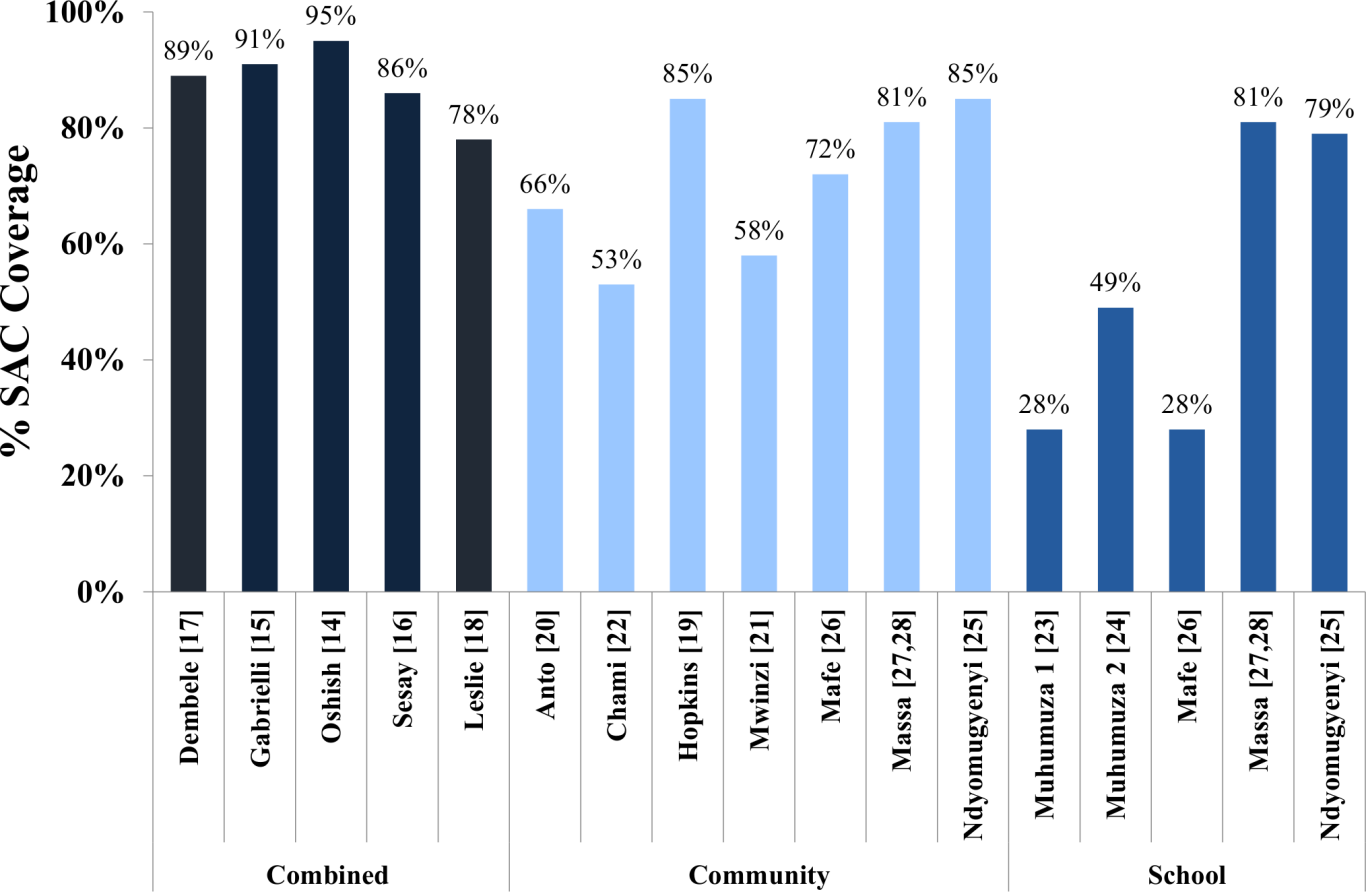
Anti-schistosomal drug treatment coverage rates by delivery method. The reported MDA coverage rates for SAC in each study are shown, grouped by delivery method (combined, community-only, or school-only). Each study is identified by its reference citation number in brackets. For the three comparative studies, both the community and school coverage rates are displayed in separate columns.

By delivery strategy, the highest SAC coverage rates were achieved with combined community- and school-based delivery (78% to 95%, median 89%) [14–18]. The second highest rates were achieved with community-only delivery, including the community arms of the comparative trials. (53% to 85%, median 72%) [19–22, 25–28]. The lowest SAC coverage rates were achieved with school-only delivery, including the school arms of the comparative trials (28% to 81%, median 49%) [23–28]. These results are shown in Fig 2.

### Coverage for non-enrolled SAC

Three studies reported separate data breakdowns on coverage rates for enrolled and non-enrolled children. In combined community/school-delivery programs, coverage results for non-enrolled children followed the trends for enrolled SAC, yielding at 90% coverage for non-enrolled SAC vs. 95% for enrolled SAC in Yemen [14] and 88% (non-enrolled) vs. 96% (in school) coverage in Burkina Faso [15]. In the comparative trial of community-based vs. school-based implementation in Tanzania, the community-based arm reached 80% non-enrolled SAC coverage [27, 28], whereas the school-based arm of the same trial reached only 59% coverage of non-enrolled-SAC [27, 28].

#### Combined community and school distribution

Five of the included programs delivered praziquantel through combined community and school channels [14–18]. In Yemen, the combined strategy was adopted due to disappointingly low coverage of non-enrolled children in a prior school-only delivery system (68% non-enrolled vs. 94% enrolled SAC coverage in 2008) [14]. After the dual channel approach was implemented in 2009, 90% coverage was achieved in non-enrolled children and 98% in enrolled children [14]. In Burkina Faso, the combined approach led to 88% coverage of non-enrolled SAC compared to 96% coverage of enrolled SAC [15], and in Sierra Leone, it led to 86% overall SAC coverage [16].

In a Mali-based study, village chiefs, community health workers, and community drug distributors (CDDs) decided on the best distribution strategy for each village, but typically it involved community-based door-to-door distribution and, if MDA occurred during the school year, school-based distribution as well. This approach achieved an overall SAC coverage rate of 89% [17].

The combined program in Niger, which reached coverage of 78%, offered treatment for children regardless of enrollment status in both the schools and communities [18].

#### Community-only distribution

There were four programs that utilized community-only distribution for MDA [19–22]. Two of the programs integrated praziquantel delivery with pre-existing onchocerciasis and lymphatic filariasis control programs, and these achieved coverage rates of 85% [19] and 66% [20], respectively. The other two programs, which were non-integrated, achieved SAC coverage rates of just 58% [21] and 53% [22].

#### School-only distribution

The only purely school-based distribution program in this review reported an initial SAC coverage rate of 28% [23]. Subsequently, interventions were put in place to increase teacher motivation and supervision in that program. A follow-up study after these interventions were implemented reported an improved SAC coverage of 49% [24].

#### Comparisons of community and school distribution

Three studies, one randomized and two non-randomized, directly compared school-based and community-based delivery [25–28]. These studies divided up delivery methods by villages or by counties. The first non-randomized study, located in Uganda, had a coverage rate of 85% in the community-based programs and of 79% in the school-based programs (p = 0.03) [25]. The other non-randomized study, located in Nigeria, achieved 72% coverage in the community-based programs and 28% in the school-based programs [26]. In the third study, which was randomized, the average coverage rate over two years was 81% in the community arm and 81% in the school arm [27, 28]. However, among non-enrolled children, coverage was significantly greater in the community arm than in the school arm: 80% versus 59% in year one, 83% versus 57% in year two, respectively (p < 0.001) [27, 28]. In this trial, non-enrolled children comprised 7.4% of the SAC population in the community-based treatment villages and 6.2% of the SAC population in the school-based treatment villages [27, 28].

### Factors associated with limited coverage

#### Fear of side effects and education/outreach efforts

In many studies, the participants, CDDs, and/or teachers reported that fear of side effects substantially impacted MDA uptake [14, 20, 23, 29, 30, 32, 36]. Side effects commonly included stomach aches, vomiting, diarrhea, headaches, and dizziness [35]. In a Ugandan study that reached only 28% MDA uptake, 72% of the children who refused praziquantel reported doing so because of fear of side effects [23]. Three hundred and thirty-seven children in that study deliberately missed school in order to avoid MDA [23]. Additionally, in an integrated program in Ghana, 18% of the people who agreed to take ivermectin and albendazole refused to take praziquantel, naming its particularly bad side effects as the reason [20].

Studies agreed that the main way to prevent severe side effects was to take praziquantel after a meal or snack [14, 34, 36]. However, many students went to school without eating, and thus were given the medication on an empty stomach. A randomized, school-based study by Muhumuza and colleagues found that providing snacks with praziquantel led to significantly higher uptake of MDA, i.e. 94% uptake in snack schools compared to 79% in non-snack schools (p = 0.002) [34].

In general, there was also a need for more education about schistosomiasis. Many studies reported gaps or misconceptions in teachers’, CDDs’, and students’ understanding of schistosomiasis [14, 23, 24, 29, 30, 32, 35]. Participants were often unconvinced of the need for treatment, particularly in the absence of symptoms and/or testing for disease [24, 32, 36]. Occasionally, false rumors were spread about the medication, e.g. that it caused cancer or that it was really an HIV/AIDS medication [29, 30, 32]. On the other hand, one study in Uganda found that correct knowledge of schistosomiasis transmission and prevention was associated with a greater likelihood of taking the treatment (adjusted odds ratio (OR) of 2.04 (CI 1.23–3.45)) [23]. In another study in Kenya, a mass media campaign was launched to explain schistosomiasis and the benefits of treatment, and CDDs confirmed that this intervention reduced anxiety in subsequent MDA deliveries [33].

#### Incentives

Eight studies addressed the issue of incentives for MDA personnel [17, 24, 26, 29–32, 36]. Lack of financial or material incentives was often identified as a constraint on MDA programs, with both CDDs and teachers desiring compensation for their involvement [26, 29, 30, 36]. In multiple cases, lack of remuneration caused low morale, poor performance, and attrition among CDDs [29–31]. Studies also frequently reported that the reason why drug distributors expected incentives was because such rewards were provided by other health programs, e.g. those for HIV, tuberculosis, and/or malaria [17, 30–32]. In one school-based program, incentives for teachers, i.e. US $2 plus t-shirts advertising the program, were introduced as part of an intervention that increased coverage among enrolled SAC from 28% to 49% [24]. Alternatively, in order to overcome distributor attrition, one study trained an especially large number of CDDs [30], and another substituted existing health facility staff for volunteer CDDs [29].

#### Ratio of distributors to recipients

Another common theme among studies was the issue of unrealistically low numbers of MDA distributors compared to recipients [14, 17, 20, 26, 31, 32, 35]. Reported ratios of distributors to recipients ranged from 1:67 to less than 1:200 [14, 17, 20, 26, 31, 32, 35]. In a Yemen-based study, the average number of people treated per two-person team per day was 203, yet the target range was set much higher, at 300 [14]. Another study calculated that the odds ratio of not receiving praziquantel was 3.4 if the CDD to population ratio was less than 1:150 [31]. The average distributor:recipient ratio in that study was 1:125 [31].

#### Gender and age

Four studies examined gender and/or age differences in coverage. One study reported that of those treated in school, 55% were male, and of those treated in the community, 51% were female. This result, according to the authors, reflected higher school enrollment rates for males [15]. The other three studies did not find a significant difference in coverage between males and females [22, 26, 28]. With respect to age, one study found that better coverage was achieved with community delivery for children ages 5–9 and with school delivery for children ages 10–14 [26].

#### Socioeconomic status

Multiple studies examined the effect of socioeconomic status on coverage [14, 22, 25, 35]. In one study in Uganda, poverty appeared to influence coverage, in that the children who went to school without a morning meal were more reluctant to take praziquantel and teachers were more reluctant to give it to them for fear of side effects [35]. Another study found increased coverage among those who had better “home quality” and among those who lived in a household that included a village council member [22]. In addition, Oshish and colleagues have argued that non-enrolled children, especially those who had withdrawn from school for financial reasons, might be too embarrassed to go to a school for treatment [14]. Another study located in Uganda found no association between coverage rates and wealth or occupation [25].

#### Ethnicity, religion, and politics

Ethnic, religious, and political affiliations were also studied for their impact on MDA coverage [21, 22, 26, 31]. In one study, the MDA campaign coincided with an election period, and political tensions affected the acceptability of CDDs in households of different political persuasions [21]. In another study, having a Muslim head of household was associated with a 52% lower likelihood of receiving PZQ compared to having a Christian head of household, despite Muslims being neither less educated nor less complaint than Christians [22]. In that same study, majority tribe members were 2.11 times more likely than minority tribe members to receive PZQ (p = 0.02) [22]. In a Nigeria-based study, the Ilaje minority group largely refused to participate in MDA [26]. Finally, in Mali, there was a significant difference (p < 0.01) in coverage between ethnic groups, with the migrant, cattle-breeding minority having a much higher rate of absenteeism during distribution [31].

## Discussion

The first aim of this systematic review was to examine the coverage rates achieved with different delivery approaches during mass drug administration (MDA) against schistosomiasis among school-age children (SAC). Among the different observational and pre-treatment/post-treatment studies included in this review, combined community- and school-based delivery achieved the highest median SAC coverage, followed by community-only delivery, then school-only delivery [14–24]. The three comparative studies found similar results, with community-based delivery either outperforming or roughly equaling school-based delivery in every case [2, 25–28]. Few studies reported specifically on coverage of non-enrolled SAC, but those that did found that offering community-based treatment (either alone or as part of a combined program) resulted in substantially better coverage for non-enrolled SAC than school-only treatment did [14, 27, 28]. However, even with community treatment, coverage was consistently lower among non-enrolled children compared to enrolled children [14, 15, 27, 28].

WHO guidelines recommend an SAC coverage rate of at least 75% in schistosomiasis-endemic areas [8]. Each of the five studies that used combined community and school distribution were able to achieve that coverage level [14–18]. On the other hand, 75% coverage was not consistently attained across the studies that used community-only or school-only delivery systems [19–28]. This suggests that for future MDA programs, combined treatment may be the most reliable choice for achieving an acceptable coverage rate.

In future studies, it will be especially important to look closely at MDA coverage for non-enrolled versus enrolled SAC, in order to ensure that all children are equitably targeted. The trend of higher coverage rates in community-based programs versus school-based programs might be explained in part by large numbers of non-enrolled children missing out on school-based distribution. If elimination of transmission is to be the eventual target for control, it is essential that there remains no persistently untreated sub-population within the community.

The second aim of this review was to identify qualitative factors affecting MDA coverage rates. On the recipient level, successful distribution was hindered by fear of medication side effects [14, 20, 23, 29, 30, 32, 36] and inadequate education about the need for MDA, despite the absence of overt symptoms [14, 23, 24, 29, 30, 32, 35]. Some of praziquantel’s gastrointestinal side effects may be minimized by giving the drug after food, and provision of porridge or other snacks may yield improved MDA uptake [14, 34, 36], and this aspect deserves greater clinical study. With regard to the efficacy of drug distributors, absence of financial or material incentives [17, 24, 26, 29–32, 36] and unrealistically high ratios of recipients to drug distributors [14, 17, 20, 26, 31, 32, 35] led to decreased motivation and staff attrition. Within communities, political tensions and differences among minority groups resulted, at times, in unequal distribution rates [21, 22, 26, 31]. On the other hand, several studies showed that snacks for recipients [34], t-shirts and small monetary rewards for teachers [24], and educational media campaigns about schistosomiasis [33] have led to more successful MDA programs. A number of the issues affecting delivery of schistosomiasis control, namely: i) limited disease knowledge, ii) fear of side effects, iii) unequal age and gender uptake, iv) lack of drug-distributor motivation and v) local political effects are also common to other MDA programs for control of NTDs. Each of these problems was noted by Krentel and colleagues [37] in their recent systematic review of factors affecting MDA participation in lymphatic filariasis elimination programs.

With this evidence in mind, it appears that small, targeted program modifications can have a meaningful positive impact on program success. Table 3 includes a listing of suggested strategies for increasing coverage rates. Implementation of these suggestions in future studies would help determine which of these adjunctive strategies, singly or in combination, lead to the most significant improvements in coverage.

**Table 3:** Specific recommendations for improving MDA coverage provided in the reviewed studies.

Goal	Suggested Strategies
**Increasing knowledge about the benefits of praziquantel **	Include education about praziquantel and schistosomiasis in drug distributor training
	Prepare staff to address common questions, e.g. “Why should I take praziquantel when I don’t have symptoms?”
	Use mass media campaigns (radio, TV, traveling road shows, posters, booklets, brochures) for education
	Conduct education sessions in village meetings, places of worship, markets, and other places where people gather
	Incorporate schistosomiasis/praziquantel education into school curricula
**Reducing fear of side effects**	Provide snacks with MDA distribution
	Schedule MDA when food is more plentiful (e.g. after a harvest)
	Educate community members about the range and transient nature of potential side effects
	Explain the link between worm burden and intensity of side effects and why side effects may thus be worse during the first round of treatment
	Have drug distributors and prominent community members publicly take praziquantel to demonstrate its safety
**Motivating and retaining drug distributors**	Increase distributor-to-recipient ratio to reduce workload
	Provide small financial or material incentives
	Avoid scheduling schistosomiasis MDA concurrently with other health programs that provide incentives
	Avoid scheduling MDA during periods of the year with especially high agricultural or other demands
**Achieving equal coverage in minority groups**	Involve all groups in the community in MDA scheduling and in distributor selection
	Monitor drug distribution in process to guard against inequities in coverage

In terms of limitations, our systematic review was limited by the small number of studies containing detailed information about schistosomiasis MDA coverage in SAC, especially with regard to non-enrolled children. Even within the eligible studies, heterogeneity in design, size, and methods used to measure and report coverage made it difficult to compare results or develop over-all estimates of the impact of individual program factors. As we have noted, there could potentially be bias in study reporting, which might then influence our current assessment. In order to compare the efficacy of different MDA control strategies more definitively, additional randomized control trials are needed, along with more consistent reporting of target population sizes and coverage rates.

Overall, this study provides a systematic first look at how to design the most effective schistosomiasis MDA programs. From a quantitative perspective, the limited data suggest that the best means to maximize SAC coverage for both enrolled and non-enrolled children is to use a combined community-based and school-based approach. Qualitative program features that are expected to maximize coverage include provision of food for treated children, educational campaigns about schistosomiasis and its treatment, increased CDD training and incentivization, and active inclusion of marginalized populations. In the push towards elimination of schistosomiasis, consideration of these factors will be essential in the development of future MDA programs. However, beyond attaining high coverage, it is also crucial to continually assure treatment efficacy through follow-up monitoring and evaluation of infection prevalence and intensity [38], and the corresponding *Schistosoma* infection-associated morbidities [39]. Optimal design of schistosomiasis surveillance strategies remains a very active area of study in operational research [40]

## Supporting information

S1 File PRISMA checklist. (DOC)

S2 File Prospero protocol CRD 42015017656 used for this study. (PDF)

S1 Text Listing of papers screened for this systematic review. (DOCX)
