# Supplementary material for: Systematic review of community-based, school-based, and combined delivery modes for reaching school-aged children in mass drug administration programs for schistosomiasis
Source: PLoS Negl Trop Dis. 2017 Oct 27;11(10):e0006043. doi: 10.1371/journal.pntd.0006043 (PMC5678727; doi:10.1371/journal.pntd.0006043)
Supplement: S2 File — (PDF) [file pntd.0006043.s002.pdf]

Experience in targeting non-enrolled school-aged children in schistosomiasis control programs

Michael Burnim, Charles King

Citation

Michael Burnim, Charles King. Experience in targeting non-enrolled school-aged children in schistosomiasis control programs. PROSPERO 2015:CRD42015017656 Available from http://www.crd.york.ac.uk/PROSPERO_REBRANDING/display_record.asp?ID=CRD42015017656

Review question(s)

What is the most effective way to reach non-enrolled school-aged children in mass drug administration (MDA) and other schistosomiasis control programs?

Searches

We will search published studies in the National Library of Medicine's MEDLINE, Elsevier's EMBASE, Google Scholar, African Journals Online, and Web of Science using key words including schistosomiasis, mansoni, haematobium, school-aged, non-enrolled, non-attendance, enrollment, community-directed treatment, coverage, compliance, and adherence.

We will search reference lists of included articles and unpublished studies will be obtained, if possible, from international schistosomiasis researchers and program directors.

Articles in English or French will be included for review, irrespective of publication date, if they include quantitative or qualitative data on treatment of non-enrolled school-aged children in sub-Saharan Africa or in other low- or middle-income countries.

Types of study to be included

No restrictions on study design although randomized studies will be weighted most heavily.

Condition or domain being studied

Schistosomiasis haematobia and schistosomiasis mansoni. Treatment coverage of total school-aged population, including non-enrolled school-aged children.

Participants/ population

Inclusion: School-aged children aged 5-15 not enrolled in school

Exclusion: School-aged children 5-15 with no data about enrollment status

Intervention(s), exposure(s)

Interventions to be reviewed include school-based treatment with or without inclusion of non-enrolled children, community directed treatment regardless of modality (house-to-house, central location, or both), and integration of schistosomiasis treatment into existing control programs (such as onchocerciasis or lymphatic filariasis).

Studies will be excluded if they only have data on enrolled school-aged children.

Comparator(s)/ control

Comparator may be routine care or comparisons may be made between different treatment modalities. Routine care is defined as an area's existing method of schistosomiasis control whether that be passive treatment, surveillance, or some form of mass treatment.

Context
Studies in population level control of schistosomiasis that target and include data on non-enrolled school-aged children. Only studies in English or French or in sub-Saharan Africa or regions with similar socioeconomic characteristics will be included.

Outcome(s)
Primary outcomes
1) Ratio of eligible non-enrolled school-aged children treated

2) Ratio of all eligible school-aged children treated

Number of eligible children treated may be measured by methods such as drug distribution record review or household survey. Total number of eligible children may be measured by methods such as household survey or community census.

Secondary outcomes
1) Where available, disease prevalence rates for the relevant populations, both before and after treatment.

2) Cost per child or case of schistosomiasis treated

3) Treatment side effects

4) Qualitative information about operational obstacles

Data extraction, (selection and coding)
Titles and/or abstracts of articles identified with our search strategy will be reviewed by two independent researchers (MB, CK) to determine if they meet eligibility criteria. If there is any ambiguity, the full article will be reviewed. Disagreements will be resolved through consensus.

Extracted data will include study information, study site, study population and participant demographics, intervention type, type of Schistosoma species, prevalence before and after intervention, cost per child treated, census and evaluation methods, coverage, side effects, and operational obstacles.

Risk of bias (quality) assessment
Risk of bias assessment is not applicable.

Strategy for data synthesis
Data synthesis methods will be dependent on the number and quality of studies identified. Aggregate data will be used to estimate coverage efficacy of each intervention type, controlling for variations in local factors such as demographics and operational obstacles such as drug shortages, cultural resistance, or difficult geography.

Analysis of subgroups or subsets
If there are sufficient data, we will perform subgroup analyses based on initial infection prevalence, type and quality of study, and study setting.

Dissemination plans
We will produce brief written materials for stakeholders of the Schistosomiasis Consortium for Operational Research and Evaluation (SCORE). We will also submit a paper for publication in an open access journal and archive data and background materials at the SCORE website.

Contact details for further information
Mr Burnim

2814 Hampshire Rd, Apt. 8

Cleveland Heights, OH 44118

United States of America

msb151@case.edu

Organisational affiliation of the review
Case Western Reserve University Center for Global Heath & Diseases

http://www.case.edu/orgs/cghd/

Review team
Mr Michael Burnim, CWRU School of Medicine
Dr Charles King, CWRU Center for Global Health and Diseases

Anticipated or actual start date
11 March 2015

Anticipated completion date
30 April 2015

Funding sources/sponsors
Schistosomiasis Consortium for Operational Research and Evaluation, University of Georgia Research Foundation

Conflicts of interest
None known

Language
English

Country
United States of America

Subject index terms status
Subject indexing assigned by CRD

Subject index terms
Child; Humans; Schistosomiasis

Stage of review
Ongoing

Date of registration in PROSPERO
06 April 2015

Date of publication of this revision
06 April 2015

DOI
10.15124/CRD42015017656

Stage of review at time of this submission	Started	Completed
Preliminary searches	Yes	No
Piloting of the study selection process	Yes	No
Formal screening of search results against eligibility criteria	No	No
Data extraction	Yes	No

Risk of bias (quality) assessment	No	No
Data analysis	No	No

PROSPERO
International prospective register of systematic reviews
The information in this record has been provided by the named contact for this review. CRD has accepted this information in good faith and registered the review in PROSPERO. CRD bears no responsibility or liability for the content of this registration record, any associated files or external websites.
