# Supplementary material for: Systematic review of community-based, school-based, and combined delivery modes for reaching school-aged children in mass drug administration programs for schistosomiasis
Source: PLoS Negl Trop Dis. 2017 Oct 27;11(10):e0006043. doi: 10.1371/journal.pntd.0006043 (PMC5678727; doi:10.1371/journal.pntd.0006043)
Supplement: S1 Text — (DOCX) [file pntd.0006043.s003.docx]

Listing of papers screened for this systematic review (included articles appear in bold)

1. Banwat ME, Ogbonna C, Daboer JC, et al. Prevalence of urinary schistosomiasis in school-aged children in Langai, Plateau State: pre- and post-intervention. Niger J Med. 2012;21(2):146-9.
2. Touré, Seydou, et al. "Two-year impact of single praziquantel treatment on infection in the national control programme on schistosomiasis in Burkina Faso." Bulletin of the world health organization 86.10 (2008): 780-787A.
3. **Gabrielli AF, Touré S, Sellin B, et al. A combined school- and community-based campaign targeting all school-age children of Burkina Faso against schistosomiasis and soil-transmitted helminthiasis: performance, financial costs and implications for sustainability. Acta Trop. 2006;99(2-3):234-42.**
4. Sangho H, Keita AD, Sacko M, Diarra Z, Simaga SY, Traore I. [Morbidity of schistosomiasis after discontinuation of mass treatment using praziquantel at a dispensary from Niger to Mali]. Med Trop (Mars). 2004;64(4):408-9.
5. King CH. Long-term outcomes of school-based treatment for control of urinary schistosomiasis: a review of experience in Coast Province, Kenya. Mem Inst Oswaldo Cruz. 2006;101 Suppl 1:299-306.
6. Mkopi A, Urassa H, Mapunjo E, Mushi F, Mshinda H. Impact of school health programme on urinary schistosomiasis control in schoolchildren in Kilosa, Tanzania. Tanzan Health Res Bull. 2005;7(3):198-200.
7. Nagi MA. Evaluation of a programme for control of schistosoma haematobium infection in Yemen. East Mediterr Health J. 2005;11(5-6):977-87.
8. Al ghahtani AG, Amin MA. Progress achieved in the elimination of schistosomiasis from the Jazan region of Saudi Arabia. Ann Trop Med Parasitol. 2005;99(5):483-90.
9. Chimbari MJ, Ndlela B. Successful control of schistosomiasis in large sugar irrigation estates of Zimbabwe. Cent Afr J Med. 2001;47(7):169-72.
10. Nsowah-nuamah NN, Mensah G, Aryeetey ME, Wagatsuma Y, Bentil G. Urinary schistosomiasis in southern Ghana: a logistic regression approach to data from a community-based integrated control program. Am J Trop Med Hyg. 2001;65(5):484-90.
11. Magnussen P, Ndawi B, Sheshe AK, Byskov J, Mbwana K, Christensen NO. The impact of a school health programme on the prevalence and morbidity of urinary schistosomiasis in Mwera Division, Pangani District, Tanzania. Trans R Soc Trop Med Hyg. 2001;95(1):58-64.
12. Talaat M, Evans DB. The costs and coverage of a strategy to control schistosomiasis morbidity in non-enrolled school-age children in Egypt. Trans R Soc Trop Med Hyg. 2000;94(4):449-54.
13. Carabin H, Chan MS, Guyatt HL. A population dynamic approach to evaluating the impact of school attendance on the unit cost and effectiveness of school-based schistosomiasis chemotherapy programmes. Parasitology. 2000;121 ( Pt 2):171-83.
14. Laamrani H, Mahjour J, Madsen H, Khallaayoune K, Gryseels B. Schistosoma haematobium in Morocco: moving from control to elimination. Parasitol Today (Regul Ed). 2000;16(6):257-60.
15. Useh MF, Ejezie GC. School-based schistosomiasis control programmes: a comparative study on the prevalence and intensity of urinary schistosomiasis among Nigerian school-age children in and out of school. Trans R Soc Trop Med Hyg. 1999;93(4):387-91.
16. Talaat M, Omar M, Evans D. Developing strategies to control schistosomiasis morbidity in nonenrolled school-age children: experience from Egypt. Trop Med Int Health. 1999;4(8):551-6.
17. Ageel AR, Amin MA. Integration of schistosomiasis-control activities into the primary-health-care system in the Gizan region, Saudi Arabia. Ann Trop Med Parasitol. 1997;91(8):907-15.
18. Magnussen P, Muchiri E, Mungai P, Ndzovu M, Ouma J, Tosha S. A school-based approach to the control of urinary schistosomiasis and intestinal helminth infections in children in Matuga, Kenya: impact of a two-year chemotherapy programme on prevalence and intensity of infections. Trop Med Int Health. 1997;2(9):825-31.
19. Montresor A, Urbani C, Camara B, Bha AB, Albonico M, Savioli L. [Preliminary survey of a school health program implementation in Guinea]. Med Trop (Mars). 1997;57(3):294-8.
20. Husein MH, Talaat M, El-sayed MK, El-badawi A, Evans DB. Who misses out with school-based health programmes? a study of schistosomiasis control in Egypt. Trans R Soc Trop Med Hyg. 1996;90(4):362-65.
21. Cline BL, Hewlett BS. Community-based approach to schistosomiasis control. Acta Trop. 1996;61(2):107-19.
22. Arinola O, Arinola A, Ojewale S. Control of urinary schistosomiasis in rural Nigeria. World Health Forum. 1996;17(3):291-2.
23. Bausch D, Cline BL. The impact of control measures on urinary schistosomiasis in primary school children in northern Cameroon: a unique opportunity for controlled observations. Am J Trop Med Hyg. 1995;53(6):577-80.
24. El malatawy A, El habashy A, Lechine N, Dixon H, Davis A, Mott KE. Selective population chemotherapy among schoolchildren in Beheira governorate: the UNICEF/Arab Republic of Egypt/WHO Schistosomiasis Control Project. Bull World Health Organ. 1992;70(1):47-56.
25. Al moagel M, Arfaa F, Abdulghani ME. The use of primary health care system in the control of schistosomiasis in the Riyadh Region of Saudi Arabia. Trop Med Parasitol. 1990;41(2):136-8.
26. Webbe G, El hak S. Progress in the control of schistosomiasis in Egypt 1985-1988. Trans R Soc Trop Med Hyg. 1990;84(3):394-400.
27. Spencer HC, Ruiz-tibén E, Mansour NS, Cline BL. Evaluation of UNICEF/Arab Republic of Egypt/WHO schistosomiasis Control Project in Beheira Governorate. Am J Trop Med Hyg. 1990;42(5):441-8.
28. Wolff T, Malewezi JG. Organization and decentralization of the Malawi National Bilharzia Control Programme. Trop Med Parasitol. 1989;40(2):201-4.
29. Savioli L, Dixon H, Kisumku UM, Mott KE. Control of morbidity due to Schistosoma haematobium on Pemba Island: programme organization and management. Trop Med Parasitol. 1989;40(2):189-94.
30. Janitschke K, Telher AA, Wachsmuth J, Jahia S. Prevalence and control of Schistosoma haematobium infections in the Amran subprovince of the Yemen Arab Republic. Trop Med Parasitol. 1989;40(2):181-4.
31. Brinkmann UK, Werler C, Traoré M, Doumbia S, Diarra A. Experiences with mass chemotherapy in the control of schistosomiasis in Mali. Trop Med Parasitol. 1988;39(2):167-74.
32. Brinkmann UK, Werler C, Traoré M, Korte R. The National Schistosomiasis Control Programme in Mali, objectives, organization, results. Trop Med Parasitol. 1988;39(2):157-61.
33. Chuks ejezie G, Okeke GC. Chemotherapy in the control of urinary schistosomiasis in Nigeria. J Trop Med Hyg. 1987;90(3):149-51.
34. Report of an independent evaluation mission on the National Bilharzia Control Program, Egypt, 1985 (abridged version). Trans R Soc Trop Med Hyg. 1987;81 Suppl:1-57.
35. Diallo S, Victorius A, Barraud BA, Ndir O. [Mass treatment of urinary bilharziasis in Senegal using praziquantel]. Dakar Med. 1984;29(1):249-54.
36. **Omedo M, Ogutu M, Awiti A, et al. The effect of a health communication campaign on compliance with mass drug administration for schistosomiasis control in western Kenya--the SCORE project. Am J Trop Med Hyg. 2014;91(5):982-8. doi: 10.4269/ajtmh.14-0136. Epub 2014 Sep 22.**
37. **Muhumuza S, Katahoire A, Nuwaha F, Olsen A. Increasing teacher motivation and supervision is an important but not sufficient strategy for improving praziquantel uptake in Schistosoma mansoni control programs: serial cross sectional surveys in Uganda. BMC Infect Dis. 2013;13:590. doi: 10.1186/1471-2334-13-590.**
38. **Omedo MO, Matey EJ, Awiti A, et al. Community health workers' experiences and perspectives on mass drug administration for schistosomiasis control in western Kenya: the SCORE Project. Am J Trop Med Hyg. 2012;87(6):1065-72. doi: 10.4269/ajtmh.2012.12-0435. Epub 2012 Oct 22.**
39. **Fleming FM, Fenwick A, Tukahebwa EM, et al. Process evaluation of schistosomiasis control in Uganda, 2003 to 2006: perceptions, attitudes and constraints of a national programme. Parasitology. 2009;136(13):1759-69. doi: 10.1017/S0031182009990709. Epub 2009 Aug 21.**
40. Talaat M, Miller FD. A mass chemotherapy trial of praziquantel on Schistosoma haematobium endemicity in Upper Egypt. Am J Trop Med Hyg. 1998;59(4):546-50.
41. Guyatt H, Evans D, Lengeler C, Tanner M. Controlling schistosomiasis: the cost-effectiveness of alternative delivery strategies. Health Policy Plan. 1994;9(4):385-95.
42. King CH, Muchiri E, Ouma JH, Koech D. Chemotherapy-based control of schistosomiasis haematobia. IV. Impact of repeated annual chemotherapy on prevalence and intensity of Schistosoma haematobium infection in an endemic area of Kenya. Am J Trop Med Hyg. 1991;45(4):498-508.
43. Tuhebwe D, Bagonza J, Kiracho EE, Yeka A, Elliott AM, Nuwaha F. Uptake of mass drug administration programme for schistosomiasis control in Koome Islands, Central Uganda. PLoS ONE. 2015;10(4):e0123673.
44. **Muhumuza S, Olsen A, Katahoire A, Nuwaha F. Uptake of preventive treatment for intestinal schistosomiasis among school children in Jinja district, Uganda: a cross sectional study. PLoS ONE. 2013;8(5):e63438.**
45. **Muhumuza S, Olsen A, Nuwaha F, Katahoire A. Understanding low uptake of mass treatment for intestinal schistosomiasis among school children: a qualitative study in Jinja district, Uganda. J Biosoc Sci. 2015;47(4):505-20.**
46. Salam RA, Maredia H, Das JK, Lassi ZS, Bhutta ZA. Community-based interventions for the prevention and control of helmintic neglected tropical diseases. Infect Dis Poverty. 2014;3:23. doi: 10.1186/2049-9957-3-23. eCollection 2014.
47. Kappagoda S, Ioannidis JP. Prevention and control of neglected tropical diseases: overview of randomized trials, systematic reviews and meta-analyses. Bull World Health Organ. 2014;92(5):356-366C.. doi: 10.2471/BLT.13.129601. Epub 2014 Mar 13
48. **Sesay S, Paye J, Bah MS, et al. Schistosoma mansoni infection after three years of mass drug administration in Sierra Leone. Parasit Vectors. 2014;7:14. doi: 10.1186/1756-3305-7-14.**
49. Leslie J, Garba A, Boubacar K, et al. Neglected tropical diseases: comparison of the costs of integrated and vertical preventive chemotherapy treatment in Niger. Int Health. 2013;5(1):78-84. doi: 10.1093/inthealth/ihs010
50. Stothard JR, Sousa-figueiredo JC, Navaratnam AM. Advocacy, policies and practicalities of preventive chemotherapy campaigns for African children with schistosomiasis. Expert Rev Anti Infect Ther. 2013;11(7):733-52. doi: 10.1586/14787210.2013.811931.
51. **Dabo A, Bary B, Kouriba B, Sankaré O, Doumbo O. Factors associated with coverage of praziquantel for schistosomiasis control in the community-direct intervention (CDI) approach in Mali (West Africa). Infect Dis Poverty. 2013;2:11-.**
52. A research agenda for helminth diseases of humans: intervention for control and elimination. PLoS Negl Trop Dis. 2012;6(4):e1549. doi: 10.1371/journal.pntd.0001549. Epub 2012 Apr 24.
53. **Dembélé M, Bamani S, Dembélé R, et al. Implementing preventive chemotherapy through an integrated National Neglected Tropical Disease Control Program in Mali. PLoS Negl Trop Dis. 2012;6(3):e1574. doi: 10.1371/journal.pntd.0001574. Epub 2012 Mar 20.**
54. **Oshish A, Alkohlani A, Hamed A, et al. Towards nationwide control of schistosomiasis in Yemen: a pilot project to expand treatment to the whole community. Trans R Soc Trop Med Hyg. 2011 Nov;105(11):617-27. doi: 10.1016/j.trstmh.2011.07.013. Epub 2011 Sep 9.**
55. Talaat M, Evans DB. The costs and coverage of a strategy to control schistosomiasis morbidity in non-enrolled school-age children in Egypt. Trans R Soc Trop Med Hyg. 2000;94(4):449-54.
56. Lillerud LE, Stuestoel VM, Hoel RE, Rukeba Z, Kjetland EF. Exploring the feasibility and possible efficacy of mass treatment and education of young females as schistosomiasis influences the HIV epidemic. Arch Gynecol Obstet. 2010;281(3):455-60. doi: 10.1007/s00404-009-1108-y. Epub 2009 May 12.
57. Talaat M, Omar M, Evans D. Developing strategies to control schistosomiasis morbidity in nonenrolled school-age children: experience from Egypt. Trop Med Int Health. 1999;4(8):551-6.
58. Useh MF, Ejezie GC. School-based schistosomiasis control programmes: a comparative study on the prevalence and intensity of urinary schistosomiasis among Nigerian school-age children in and out of school. Trans R Soc Trop Med Hyg. 1999;93(4):387-91.
59. Community-directed interventions for priority health problems in Africa: results of a multicountry study. Bull World Health Organ. 2010;88(7):509-18.
60. Ndyomugyenyi R, Kabali AT. Community-directed interventions for integrated delivery of a health package against major health problems in rural Uganda: perceptions on the strategy and its effectiveness. Int Health. 2010;2(3):197-205.
61. **Massa K, Magnussen P, Sheshe A, Ntakamulenga R, Ndawi B, Olsen A. Community perceptions on the community-directed treatment and school-based approaches for the control of schistosomiasis and soil-transmitted helminthiasis among school-age children in Lushoto District, Tanzania. J Biosoc Sci. 2009;41(1):89-105.**
62. **Hopkins DR, Eigege A, Miri ES, et al. Lymphatic filariasis elimination and schistosomiasis control in combination with onchocerciasis control in Nigeria. Am J Trop Med Hyg. 2002;67(3):266-72.**
63. **Anto F, Asoala V, Anyorigiya T, et al. Simultaneous administration of praziquantel, ivermectin and albendazole, in a community in rural northern Ghana endemic for schistosomiasis, onchocerciasis and lymphatic filariasis. Trop Med Int Health. 2011;16(9):1112-9.**
64. Hodges ME, Koroma JB, Sonnie M, Kennedy N, Cotter E, Macarthur C. Neglected tropical disease control in post-war Sierra Leone using the Onchocerciasis Control Programme as a platform. Int Health. 2011;3(2):69-74.
65. **Mwinzi PN, Montgomery SP, Owaga CO, et al. Integrated community-directed intervention for schistosomiasis and soil transmitted helminths in western Kenya - a pilot study. Parasit Vectors. 2012;5:182.**
66. Makaula P, Bloch P, Banda HT, et al. Primary Health Care in rural Malawi - a qualitative assessment exploring the relevance of the community-directed interventions approach. BMC Health Serv Res. 2012;12:328.
67. **Chami GF, Kontoleon AA, Bulte E, et al. Profiling Nonrecipients of Mass Drug Administration for Schistosomiasis and Hookworm Infections: A Comprehensive Analysis of Praziquantel and Albendazole Coverage in Community-Directed Treatment in Uganda. Clin Infect Dis. 2016;62(2):200-7.**
68. Tadesse Z, Hailemariam A, Kolaczinski JH. Potential for integrated control of neglected tropical diseases in Ethiopia. Trans R Soc Trop Med Hyg. 2008;102(3):213-4.
69. Kaatano GM, Siza JE, Mwanga JR, et al. Integrated Schistosomiasis and Soil-Transmitted Helminthiasis Control over Five Years on Kome Island, Tanzania. Korean J Parasitol. 2015;53(5):535-43.
70. Lo NC, Bogoch II, Blackburn BG, et al. Comparison of community-wide, integrated mass drug administration strategies for schistosomiasis and soil-transmitted helminthiasis: a cost-effectiveness modelling study. Lancet Glob Health. 2015;3(10):e629-38.
71. Colley DG. Morbidity control of schistosomiasis by mass drug administration: how can we do it best and what will it take to move on to elimination?. Trop Med Health. 2014;42(2 Suppl):25-32.
72. Lelo AE, Mburu DN, Magoma GN, et al. No apparent reduction in schistosome burden or genetic diversity following four years of school-based mass drug administration in mwea, central kenya, a heavy transmission area. PLoS Negl Trop Dis. 2014;8(10):e3221.
73. Humphries D, Nguyen S, Boakye D, Wilson M, Cappello M. The promise and pitfalls of mass drug administration to control intestinal helminth infections. Curr Opin Infect Dis. 2012;25(5):584-9.
74. Hodges MH, Dada N, Warmsley A, et al. Mass drug administration significantly reduces infection of Schistosoma mansoni and hookworm in school children in the national control program in Sierra Leone. BMC Infect Dis. 2012;12:16.
75. Parker M, Allen T. Does mass drug administration for the integrated treatment of neglected tropical diseases really work? Assessing evidence for the control of schistosomiasis and soil-transmitted helminths in Uganda. Health Res Policy Syst. 2011;9:3.
76. **Ndyomugyenyi R, Kabatereine N. Integrated community-directed treatment for the control of onchocerciasis, schistosomiasis and intestinal helminths infections in Uganda: advantages and disadvantages. Trop Med Int Health. 2003;8(11):997-1004.**
77. **a. Massa K, Magnussen P, Sheshe A, Ntakamulenga R, Ndawi B, Olsen A. The effect of the community-directed treatment approach versus the school-based treatment approach on the prevalence and intensity of schistosomiasis and soil-transmitted helminthiasis among schoolchildren in Tanzania. Trans R Soc Trop Med Hyg. 2009;103(1):31-7.**

**b. Massa K, Olsen A, Sheshe A, Ntakamulenga R, Ndawi B, Magnussen P. Can coverage of schistosomiasis and soil transmitted helminthiasis control programmes targeting school-aged children be improved? New approaches. Parasitology. 2009;136(13):1781-8.**

1. Saathoff E, Olsen A, Magnussen P, Kvalsvig JD, Becker W, Appleton CC. Patterns of Schistosoma haematobium infection, impact of praziquantel treatment and re-infection after treatment in a cohort of schoolchildren from rural KwaZulu-Natal/South Africa. BMC Infect Dis. 2004;4:40.
2. Olsen A. Efficacy and safety of drug combinations in the treatment of schistosomiasis, soil-transmitted helminthiasis, lymphatic filariasis and onchocerciasis. Trans R Soc Trop Med Hyg. 2007;101(8):747-58.
3. Webbe G, El hak S. Progress in the control of schistosomiasis in Egypt 1985-1988. Trans R Soc Trop Med Hyg. 1990;84(3):394-400.
4. Brooker S, Kabatereine NB, Fleming F, Devlin N. Cost and cost-effectiveness of nationwide school-based helminth control in Uganda: intra-country variation and effects of scaling-up. Health Policy Plan. 2008;23(1):24-35.
5. **Kabatereine NB, Tukahebwa E, Kazibwe F, et al. Progress towards countrywide control of schistosomiasis and soil-transmitted helminthiasis in Uganda. Trans R Soc Trop Med Hyg. 2006;100(3):208-15.**
6. Montresor, A. Helminth control in school age children: a guide for managers of control programmes - 2nd ed. World Health Organization, 2012.
7. **Parsitological Impact of 2-year preventive chemotherapy on schistosomiasis and soil-transmitted helminthiasis in Uganda**
8. Schistosoma mansoni in school attendees and non-attendees in Northwest Ethiopia. The Ethiopian Lournal of Health Development 15, 117–123. (2001)
9. Extending anthelminthic coverage to non-enrolled school-age children using a simple and low-cost method
10. Evans DB, Guyatt HL. The cost effectiveness of mass drug therapy for intestinal helminths. Pharmacoeconomics. 1995;8(1):14-22.
11. Kabatereine NB, Brooker S, Koukounari A, et al. Impact of a national helminth control programme on infection and morbidity in Ugandan schoolchildren. Bull World Health Organ. 2007;85(2):91-9.
12. Garba A, Touré S, Dembelé R, Bosque-oliva E, Fenwick A. Implementation of national schistosomiasis control programmes in West Africa. Trends Parasitol. 2006;22(7):322-6.
13. Kabatereine NB, Fleming FM, Nyandindi U, Mwanza JC, Blair L. The control of schistosomiasis and soil-transmitted helminths in East Africa. Trends Parasitol. 2006;22(7):332-9.
14. Brooker S, Whawell S, Kabatereine NB, Fenwick A, Anderson RM. Evaluating the epidemiological impact of national control programmes for helminths. Trends Parasitol. 2004;20(11):537-45.
15. Progress in the control of schistosomiasis in Egypt during the past five years 1988-1992. Cairo: Ministry of Heatlh, Egypt (Report). (El-Kohby, Webbe) 1992.
16. El-Khoby, T., El-Zeimaty, 0 & Fenwick, A. (1991). Countries where both Schistosoma haematobium and S. mansoni are endemic: progress in control of schistosomiasis since 1984 in Egypt. Geneva: World Health Organization, mimeographed document Sch/EC/Wp. 42.
17. Evans. D. B. & Guvatt, H. L. (1995). The cost-effectiveness of mass drug therapy’for intestinal helminths. Pharmaco-Economics, 8, l-8.
18. Nokes, C. & Bundy, D. A. I’. (1993). Compliance and absenteeism in school children: implications for helminth control. Transactions of the Royal Society of Tropical Medicine and Hygiene, 87, 148-152.
19. WHO (1993). The Control of Schistosomiasis. Geneva: World Health Organization, Technical Report Series, no. 830.
20. Albonico, M., Crompton, D.W.T., Savioli, L., 1999. Control strategies for human intestinal nematode infections. Adv. Parasitol. 42, 277—341.
21. Katabereine, N.B. 2000. Schistosoma mansoni in a fishing community on the shores of Lake Albert at Butiaba, Uganda. Epidemiology, morbidity, re-infection pattenrs, and impact of treatment with praziquantel. PhD Thesis, Faculty of Science, University of Copenhagen, Denmark.
22. Kabatereine NB, Tukahebwa EM, Kazibwe F, et al. Soil-transmitted helminthiasis in Uganda: epidemiology and cost of control. Trop Med Int Health. 2005;10(11):1187-9.
23. Ageel, A.R., Amin, M.A., 1997. Integration of schistosomiasiscontrol activities into primary health-care system in the Gizan region, Saudi Arabia. Ann. Trop. Med. Parasitol. 91, 907–915.
24. Engels, D., Chitsulo, L., Montressor, A., Savioli, L., 2002. The global epidemiological situation of Schistosomiasis and new approaches to control and research. Acta Trop. 2, 136–146.
25. Partnership for Child Development (PCD), 1999. The cost of largescale school health programmes which deliver anthelmintics to children in Ghana and Tanzania. Acta Trop. 73, 183–204
26. WHO Geneva, 2001. Schistosomiasis and Soil-transmitted Helminth Infection. Executive Board Resolution EB107/31.
27. Ndyomugyenyi R, Minjas JN. Urinary schistosomiasis in schoolchildren in Dar-es-Salaam, Tanzania, and the factors influencing its transmission. Ann Trop Med Parasitol. 2001;95(7):697-706.
28. Webbe G. Community-wide treatment of schistosomiasis with praziquantel. Trop Doct. 1999;29(3):172-6.
29. Control of geohelminths by delivery of targeted chemotherapy through schools. Transactions of the Royal Society of Tropical Medicine and Hygiene 84, 115–120. (1990)
30. Different Approaches to Modeling The Cost-Effectiveness of Schistosomiasis Control. American Journal of Tropical Medicine and Hygiene 55, 159–164. (1996)
31. Cost analysis of a school-based drug delivery programme in Tanzania. Oxford: Cientific Co-ordinating Centre of the Partnership for Child Development, Univ of Oxford, UK. (1997b)
32. Geographical distribution of intestinal schistosomiasis and soil-transmitted helminthiasis and preventive chemotherapy strategies in Sierra Leone. PLoS Negl Trop Dis. 2010 Nov 23;4(11):e891. doi: 10.1371/journal.pntd.0000891. (2010)
33. Peter, Jordan; Webbe, Gerald; Sturrock Robert F. Human Schistosomiasis. Wallingford, Oxon, UK : CAB International, c1993.
34. Hammad TA, Gabr NS, Hussein MH, Orieby A, Shawky E, Strickland GT. Determinants of infection with schistosomiasis haematobia using logistic regression. Am J Trop Med Hyg. 1997;57(4):464-8.
35. **Leslie J, Garba A, Oliva EB, Barkire A, Tinni AA, Djibo A, et al. Schistosomiais and soil-transmitted helminth control in Niger: cost effectiveness of school based and community distributed mass drug administration. PLoS Negl Trop Dis. 2011;5(10):e1326. doi: 10.1371/journal.pntd.0001326.**
36. **Muhumuza S, Olsen A, Katahoire A, Kiragga AN, Nuwaha F. Effectiveness of a pre-treatment snack on the uptake of mass treatment for schistosomiasis in Uganda: a cluster randomized trial. PLOS Medicine. 2014;11(5):e1001640.**
37. **Mafe MA, Appelt B, Adewale B, Idowu ET, Akinwale OP, Adeneye AK, et al. Effectiveness of different approaches to mass delivery of praziquantel among school-aged children in rural communities in Nigeria. Acta Tropica. 2005;93(2):181-90.**
